# Current Antimycotics, New Prospects, and Future Approaches to Antifungal Therapy

**DOI:** 10.3390/antibiotics9080445

**Published:** 2020-07-25

**Authors:** Gina Wall, Jose L. Lopez-Ribot

**Affiliations:** Department of Biology and The South Texas Center for Emerging Infectious Diseases, The University of Texas at San Antonio, One UTSA Circle, San Antonio, TX 78249, USA; gina.wall@utsa.edu

**Keywords:** fungal infections, antifungal agents, antifungal drug development

## Abstract

Fungal infections represent an increasing threat to a growing number of immune- and medically compromised patients. Fungi are eukaryotic organisms and, as such, there is a limited number of selective targets that can be exploited for antifungal drug development. This has also resulted in a very restricted number of antifungal drugs that are clinically available for the treatment of invasive fungal infections at the present time—polyenes, azoles, echinocandins, and flucytosine. Moreover, the utility of available antifungals is limited by toxicity, drug interactions and the emergence of resistance, which contribute to high morbidity and mortality rates. This review will present a brief summary on the landscape of current antifungals and those at different stages of clinical development. We will also briefly touch upon potential new targets and opportunities for novel antifungal strategies to combat the threat of fungal infections.

## 1. Fungi and Fungal Infections

It has been estimated that there are up to 5 million fungal species populating the earth today [1], of which approximately 100,000 species have been identified. Interestingly, to date, only about 300 fungal species have been described to cause disease in humans. Fungi that infect humans must satisfy a set of four particular characteristics: they must have the ability to grow at or above 37 °C; they must have the ability to reach the tissues associated with their pathogenesis; they must be able to digest and absorb elements of human tissues for their own nutrition; and lastly they must withstand the human immune system [2]. Those fulfilling these criteria are able to cause a variety of infections that range from skin and mucosal infections to life-threatening invasive infections, with less than 30 species causing over 99% of infections [3]. Although a few fungal species are fully capable of causing infections in healthy immunocompetent individuals, the majority of them are opportunistic pathogens, requiring a susceptible host [4]. Thus, besides the recent HIV epidemic, the major contributing factor for the increase in fungal infections has been the fact that advances in modern medicine and the successful treatment of serious underlying diseases have created an ever-expanding population of immunosuppressed and medically compromised patients who are inherently susceptible to these devastating infections [4].

Major risk factors for the development of fungal infections include, among others, cytotoxic chemotherapy in cancer patients, immunosuppressive therapy in transplant patients, broad-spectrum antibiotics, trauma, burn, surgical and intensive care unit (ICU) patients where innate defenses have been breached, diabetes, parenteral nutrition, extremes of age (very-low-birth-weight neonates and the elderly), and the presence of catheters and other indwelling devices. Overall, *Candida*, *Cryptococcus* and *Aspergillus* represent the main causative organisms of invasive fungal infections [4]; however infections caused by other emerging yeasts (exemplified by the recent global spread of *Candida auris*) and moulds (i.e., the mucorales, Fusarium spp, Scedosporium spp, etc.), which are often multi-drug resistant, are on the rise, with a nearly 10-fold increase in reports of newly described fungal human pathogens since 1995 [5]. 

In addition, fungal pathogens have nefarious consequences to plant and animal life on our planet, being directly responsible for the destruction of a third of all food crops each year (estimated to be enough to feed over 600 million people), as well as for the recently observed mass extinctions of amphibians and mass mortality of bees and bats that pose a significant threat to the overall biodiversity of our planet [5]. Despite these facts, fungal infections have traditionally received much less attention than other types of infections, such as those caused by bacteria, viruses and parasites; because of the lack of concomitant public awareness, fungi and fungal infections have recently been referred to as the “hidden killers/the neglected epidemic” [6,7].

## 2. Current Antifungal Drugs

**Figure 1:**
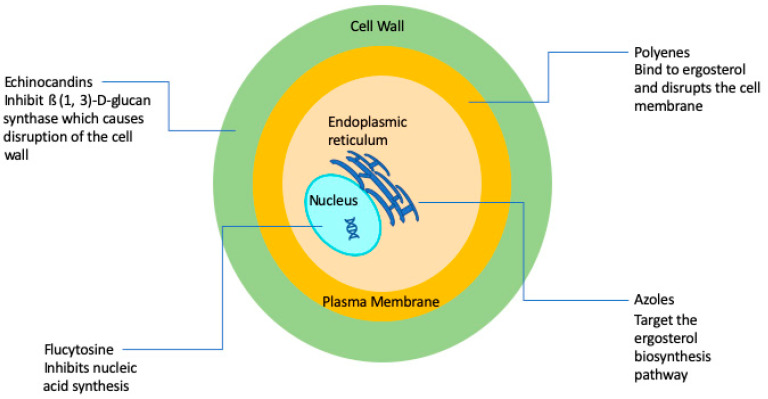
Simplified schematic diagram depicting the current antifungals and their modes of action.

A major factor contributing to the high levels of morbidity and mortality of fungal infections is the limited number of antifungal drugs [8]. Fungi, like humans, are eukaryotic organisms and there is a paucity of selective targets that can be exploited for antifungal drug development. As a consequence, and in stark contrast with antibacterials, the current antifungal armamentarium is still very limited [8,9]. The 1990s can be considered the “golden era” of antifungal drug development with multiple big pharmaceutical companies actively engaged in the discovery and development of novel antifungals. However, antifungal drug development has largely become stagnant since then, and it has been now two decades since the newest class of antifungal agents (the echinocandins) reached the market. Overall, there are currently four classes of FDA-approved antifungal agents clinically used in the treatment of invasive fungal infections, namely the polyenes, flucytosine, the azoles, and the echinocandins [10] (Figure 1).

The polyenes were the first broad-spectrum antifungals available for use in humans with their discovery in the 1940s and 1950s [10], with Amphotericin B representing the first ever FDA-approved antifungal for the treatment of invasive fungal infections [10], and for many years remained the gold standard for the therapy of mycoses. The amphipathic Amphotericin B molecule binds to ergosterol and also acts as a “sponge” that extracts sterols from the cell membranes of fungi [10,11,12,13], leading to a weakened membrane and leakage of cytosolic contents, ultimately leading to cell death. Therefore, polyenes are considered to be fungicidal and display a broad spectrum of activity against most fungal organisms. The major drawback of amphotericin B has always been its toxicity, particularly nephrotoxicity that can lead to kidney failure. Thus, a number of lipid formulations of amphotericin B, including liposomal amphotericin B (LAmB), amphotericin B lipid complex (ABLC), and amphotericin B colloidal dispersion (ABCD) have been developed, which generally show decreased toxicity and improved pharmacokinetics, which depend to a large extent on the composition and particle size of the nanoformulations [14]. For example, LAmB is composed of small unilamellar particles (true liposomes), resulting in high serum and CNS concentrations, whereas ABLC and ABCD typically achieve much higher levels in organs of the reticuloendothelial system [14]. A major drawback of these formulations is that they are significantly more expensive than conventional amphotericin B. In general, resistance to amphotericin B is rare, although some fungal species display intrinsic resistance to polyenes [15,16].

The antifungal 5-fluorocytosine was first synthesized in 1957 as a potential anti-tumor agent, and it was approved for use in humans as an antifungal in 1968 [17,18]. Flucytosine is a pyrimidine, known to inhibit DNA and RNA synthesis in fungi because of its ability to be metabolized to 5-flourouracil that is then incorporated into RNA [19]. A major problem with flucytosine is the extremely common occurrence of the development of resistance, and, because of this, it is never used in monotherapy, but rather in combination with other antifungals [20]. In addition, hepatotoxicity and hematological toxicity are common adverse effects at target concentrations [17]. Its clinical use is mostly restricted to the treatment of cryptococcal meningitis [17,21].

The first azole was discovered in 1944, but azoles were not approved for use in humans until the late 1950s/early 1960s [10]. Azoles inhibit 14-α-sterol demethylase, a cytochrome P-450 enzyme involved in the synthesis of ergosterol [22], which results in the accumulation of toxic sterol intermediaries and loss of membrane integrity. Most azoles are fungistatic and have a relatively broad spectrum of activity against yeasts and filamentous fungi [21,23]. The introduction of triazoles in the clinics during the 1980s and 1990s revolutionized the treatment of fungal infections [24,25]. The most common azole used in systemic fungal infections, particularly those caused by yeasts (i.e., *Candida*, *Cryptococcus*), is fluconazole, which was released in 1990. However, some fungal species, including *Aspergillus* and other emerging moulds, display intrinsic (or primary) resistance to fluconazole, and other derivatives were developed that display activity against *Aspergillus* (i.e., itraconazole, voriconazole) and those with an expanded spectrum of activity that includes the mucorales (i.e., posaconazole and most recently isavuconazole). Due to their fungistatic nature, the development of resistance is a common occurrence during treatment with azoles, mostly through mutations in the target gene *ERG11*, but also because of the overexpression of efflux pumps that transport the azole molecules outside the cell [15]. In spite of resistance, azoles are still some of the most commonly used antifungal drugs for the treatment of a variety of fungal infections.

The echinocandins are the most recent class of antifungals that have been approved by the FDA [21,26]. They were first discovered in the 1970s but did not enter the market until the early 2000s [27]. They target 1,3-β-d-glucan synthase, an enzyme responsible for the production of 1,3-β-d-glucan, a polysaccharide which constitutes the main structural component of most fungal cell walls. Caspofungin was the first echinocandin to be approved for use in humans in 2001 [28,29,30]. Two other echinocandins were subsequently produced and approved: micafungin in 2005 and anidulafungin in 2006 [29,30,31]. Because mammalian cells do not have a cell wall, their target allows for significantly less toxicity and drug–drug interactions as compared to other antifungals [32,33]. These drugs were originally approved for treatment of aspergillosis infections that were refractory to treatment with other antifungals [30,34,35], but they have since been approved for the treatment of candidiasis for which they are now considered the agents of choice, as well as for other fungal infections [30]. Although resistance has been relatively rare, most recently, as a consequence of increased drug exposure, there has been an increase in development of resistance mostly in non-*albicans Candida* spp. [29]. The main mechanism of resistance against echinocandins is mutations in the *FKS1* and *FKS2* encoding the target enzyme [15,36,37]. Echinocandins are considered fungicidal and effective against a majority of *Candida* spp. [30], but fungistatic against *Aspergillus* spp. [38]. However, the spectrum of action of echinocandins does not include *Cryptococcus* and some of the emerging pathogens [32,38].

Table 1 below summarizes the main characteristics of these classes of antifungal agents.

## 3. An Update on the Antifungal Drug Discovery and Development Pipeline

Currently, the antifungal pipeline in big pharmaceutical companies is mostly dried, as the focus of these companies is mainly on high-profit drugs for the treatment of chronic diseases associated with our sedentary lifestyle. As a result, antifungal research and development (R&D) has been discontinued at most pharmaceutical firms, which are looking to smaller biotech companies with more limited resources to supply the next generation of novel antifungal drugs due to their own decreasing investment in antibiotic R&D. Compared to other anti-infectives, there are relatively few companies involved in antifungal R&D. However, in the United States, the fact that both the GAIN (Generating Antibiotic Incentives Now) and Orphan Drug Acts, as well as the Fast Track designation by the Food and Drug Administration, all potentially apply to new antifungal agents has led to a renewed interest in antifungal drug development.

Several investigational agents are currently under development. Of note, most of these also target either ergosterol (same as azoles) or 1,3-β-d-glucan (same as echinocandins). Among these are the tetrazoles VT-1129, VT-1161, and VT-1598, which, compared to triazoles, are more specific for fungal Cyp51 and less so for mammalian CYP 450 enzymes [39,40,41]; the echinocandin rezafungin (formerly CD101) that has an extended half-life [42]; and the glucan synthase inhibitor Ibrexafungerp (formerly SCY-078), which is being developed for oral administration [43]. In addition, several agents with novel targets and mechanisms of action are also under development. These include fosmanogepix, formerly APX001, which inhibits the inositol acyltransferase Gwt1 in the glycosylphosphatidylinositol (GPI) anchor biosynthesis pathway, thereby preventing GPI-anchored protein maturation [44]; the dihydroorotate dehydrogenase (an enzyme of the de novo pyrimidine biosynthesis) inhibitor, F901318 (Olorofim) [45]; and VL-2397, which is similar in structure to the siderophore ferrichrome [46]. Moreover, although first discovered in the 1970s, the chitin inhibitor Nikkomycin Z is currently undergoing clinical trials against coccidioidomycosis [47]. Other notable molecules under development include Aureobasidin A, a cyclic peptide that inhibits fungal sphingolipid biosynthesis with potent broad spectrum antifungal activity [48]; and T-2307, from Toyama Corporation, an investigational arylamidine, which is active against yeast and moulds [49].

## 4. Future Targets and Alternative Approaches

In the case of current clinically used antifungal agents, new formulations and delivery systems can, in principle, allow for the development of targeted therapies, which should lead to enhanced efficacy and reduced toxicity, resulting in overall improved patient outcomes. The same is true for the application of antifungal pharmacokinetic/pharmacodynamic (PK/PD) principles, where better in vitro and in vivo PK/PD models, more accurate clinical antifungal PK/PD predictions, and therapeutic monitoring may lead to optimized antifungal dosing and increase the probability of a successful outcome for patients with fungal infections. Readers are referred to excellent reviews on these topics [50,51].

Of course, the basic research currently being conducted mostly in academic settings may provide important information on potential fungal-specific pathways and novel antifungal targets as well as pave the way in the near future for the development of new antifungal drugs with new molecular structures and mechanisms of action. Perhaps among the most promising targets to date are the calcineurin and RAS pathways, sphingolipid biosynthesis, as well as the trehalose biosynthetic pathway. This topic has been reviewed recently in an excellent and rather insightful publication [3].

Moreover, research on the mechanisms of fungal pathogenesis over the past two–three decades, and in particular during the post-genomic era, may lead to the development of novel anti-virulence agents, which exert their activity by disarming the fungal cells of their virulence properties as opposed to inhibiting their growth, and may serve as the basis for alternative approaches for the treatment of fungal infections [52,53]. This is a particularly attractive alternative to combat infections caused by opportunistic pathogenic fungi. One of the main advantages of anti-virulence approaches is that they automatically expand the number of potential antifungal targets. Moreover, because of lower selective pressure, anti-virulence approaches have lower propensity to elicit antifungal drug resistance. *C. albicans* has been the organism for which more work on this topic has been performed to date, with filamentation and biofilm formation, two of the most important virulence factors inextricably linked to the pathogenesis of candidiasis, having been identified as high-value targets for the development of such novel anti-virulence strategies [54,55].

The last decade has also witnessed an increased interest in repurposing as a potential accelerated path towards antifungal drug development. In contrast to the arduous, long and very expensive process of de novo drug development, repurposing (also referred to as repositioning) involves the investigation of novel therapeutic indications for existing drugs. Repurposing allows for the identification of drugs with known pharmacokinetics, pharmacodynamics, and safety levels in humans, which, in turn, makes this approach less costly, less time-consuming, and more likely to succeed than traditional de novo discovery. As such, repurposing efforts can lead to the quick deployment of novel antifungal drugs and significantly shorten the transition from bench to bedside. This could be particularly important in case of multi-drug-resistant and emerging pathogens, as exemplified by the rapid spread of *C. auris* infections in the last few years. Many examples of research aimed at repurposing existing drugs as antifungals can be found in the literature [56,57,58,59,60,61]. Most recently, these efforts have taken advantage of the availability of repurposing libraries, a collection of hundreds of existing drugs, that can be conveniently screened for the identification of drugs with novel antifungal activity [62,63,64,65,66,67,68,69,70].

Finally, we would like to highlight the use of nanotechnology, using nanoscale materials, as an alternative strategy for antifungal drug development, which has been gaining traction over the last few years [71]. These so called “nanomaterials” or “nanoantibiotics” can be described as single structures, the size of which is less than 100 nm in at least one of their three dimensions. The increasing interest in nanomaterials is due to their novel or improved physicochemical properties, [72] such as endurance, chemical reactivity, biocompatibility, conductivity, and reduced toxicity. Although a variety of nanomaterials have been evaluated, the majority of research on this topic has used metal nanoparticles, synthesized by different methods, and evaluated their direct antifungal activity [73,74]. Of these, silver nanoparticles have received the most attention, as the antimicrobial properties of silver has been known for centuries. The increased antifungal activity is generally associated with the smaller size of the nanoparticles, with shape and surface area also playing a major role. Importantly, nanoparticles have been described to display potent activity against drug-resistant fungal biofilms [75,76].

## 5. Conclusions

The arsenal of antifungal drugs currently available to treat fungal infections is limited, contributing to high morbidity and mortaltiy rates. Problems with toxicity and emergence of resistance to clinically-used antifungals further complicates treatment and leads to poor outcomes in patients. Novel antifungal agents in the development pipeline and alternative approaches that are presently being investigated would seem to point to a bright future for antifungal drug development.

## Figures and Tables

**Table 1:** The four main classes of clinically-used antifungal agents for the treatment of invasive fungal infections.

Antifungal Class	Mechanism of Action	Biological Effect	Spectrum of Action
Polyenes	Target ergosterol and extract sterols from fungal cell membranes	Fungicidal	Broad spectrum antifungal in treatment of invasive fungal infections; resistance is rare
Flucytosine	Inhibits DNA and RNA synthesis	Fungicidal against *Cryptococcus* spp.	Almost exclusively used for cryptococcal meningitis, but resistance is extremely common so never used in monotherapy
Azoles	Inhibit 14-α-lanosterol demethylase thereby inhibiting ergosterol synthesis	Mostly fungistatic	As a class they display broad spectrum against yeasts and filamentous fungi, although some species display intrinsic resistance to commonly used derivatives; secondary resistance can often develop during treatment
Echinocandins	Target 1,3-β-d-glucan synthase, thus preventing production of cell wall 1,3-β-d-glucan	Fungicidal against *Candida* spp., but fungistatic against *Aspergillus* spp.	First line of defense for candidiasis and used in aspergillosis when refractory to other treatments; resistance is emerging
